# Functional Neurological Symptom Disorder: A Continuing Conundrum for the Perioperative Physician

**DOI:** 10.7759/cureus.42011

**Published:** 2023-07-17

**Authors:** Phillip Chin, Sangeeta Kumaraswami

**Affiliations:** 1 Anesthesiology, New York Medical College/Westchester Medical Center, Valhalla, USA

**Keywords:** functional neurological syndrome, psychiatric, conversion disorder, functional neurological disorder, functional neurological symptom disorder

## Abstract

Functional neurological symptom disorder (FNSD) or functional neurological disorder (FND) or conversion disorder, is a syndrome of neurological complications unexplained by neuropathology. The term FNSD or FND is now preferred, as conversion disorder is not an etiologically neutral term and is thus falling from use by researchers and clinicians in the field.

We report a case of new-onset postoperative neurological deficit in a patient who had undergone uneventful general anesthesia for a urology procedure. Postoperatively, in the post-anesthesia care unit, the patient was found to be unable to move her upper and lower limbs. Organic pathology was excluded and a diagnosis of FNSD was made. Four weeks after the surgery, the patient was only able to ambulate with the help of a mechanical walker device.

It is now suggested that procedures involving anesthesia are relatively common triggers for the development of FNSD. The occurrence of FNSD in the postoperative period is increasingly being attributed to the effects of anesthesia, the hypothesis being that it arises from the abreactive or dissociative effects of anesthetic agents. Another theory is the vulnerability of the anesthetized state which may evoke previous traumatic experiences. Psychiatric co-morbidities such as anxiety and depression may be seen in these patients. Preoperative psychological assessment may help identify patients at risk for FNSD. If postoperative neurological deficit occurs, detailed neurological, metabolic, and psychiatric assessments should be done with FNSD being a diagnosis of exclusion. We present this case to increase awareness regarding this uncommon condition which can cause significant distress to the patient and healthcare team. Management should comprise honest disclosure, reassurance of recovery, and reinforcement of alternative coping strategies. The development of preoperative screening tools may help identify patients at risk for this disorder.

## Introduction

Functional neurological symptom disorder (FNSD) or functional neurological disorder (FND) or conversion disorder, is a syndrome of neurological complications unexplained by neuropathology [1]. It is described as the occurrence of neurological symptoms due to malfunction, rather than neuropathology or neurological disease of the nervous system [2]. Conversion disorder is not an etiologically neutral term and is currently falling from use by researchers and clinicians in the field [3].

Current diagnostic criteria for FNSD include: a) One or more symptoms of altered voluntary motor or sensory function. b) Clinical findings that show evidence of incompatibility between the symptoms and recognized neurological or medical conditions. c) Symptoms or deficits that are not better explained by another medical or mental disorder. d) Symptoms or deficits that cause clinically significant distress or impairment in social, occupational, or other important areas of functioning or warrant medical evaluation [4].

A recent review of patients who had developed FNSD within 48 hours of anesthesia, concluded that there is support for an etiological role for anesthesia [1]. The review suggested that procedures involving anesthesia were relatively common triggers for the development of FNSD. FNSD has been described after moderate sedation, neuraxial anesthesia, and general anesthesia [5-10]. Deficits that have been reported include monoplegia, hemiplegia, paraplegia, quadriplegia, amnesia, aphonia, delayed awakening, blindness, deafness, and seizures. It has been contemplated whether anesthetic agents or the anesthetized state predispose a vulnerable brain to manifest these symptoms and signs [1,5,6]. Although the existence of psychological factors, such as conflicts or stress, are common and judged to be associated with the deficits [4], they are not universally present [2].

We present our case to increase awareness regarding this uncommon disorder which can present unique challenges.

## Case presentation

A 63-year-old woman presented for ambulatory surgery for placement of a midurethral sling for urinary incontinence. Her medical history was significant for breast cancer. Her surgical history included two breast surgeries, hysterectomy, and colonoscopies. She reported delayed awakening and postoperative nausea and vomiting with some of her previous surgeries and was anxious about a similar experience with this surgery. Within the last year, she had undergone a laparoscopic surgery and a colonoscopy with an uneventful induction and emergence at our hospital. She was counseled that drowsiness after general anesthesia may be confused for delayed awakening. She was reassured that she would receive prophylactic medications to prevent postoperative nausea and vomiting. She declined spinal anesthesia for the surgery.

The patient received an intravenous anxiolytic before going into the operating room. General anesthesia was induced and a supraglottic airway was placed without complications. The urology procedure was performed in the lithotomy position. Intraoperatively, the patient remained hemodynamically stable. The total operative time was one-and-a-half hours. Emergence was uneventful with the supraglottic airway being removed soon after completion of surgery. She was transferred awake to the post-anesthesia care unit.

One hour later, it was noticed that the patient was unable to move her bilateral upper and lower extremities. The patient was able to speak and appropriately communicate. Physical examination showed intact sensation and reflexes in both the upper and lower extremities. Vital signs and laboratory parameters were unremarkable. A neurology consultation was requested. A hand raise drop test was done which showed that when the patient’s arm was raised and then released, it did not fall on the patient’s face but behind her head or above her head. A computed tomography scan of the head and cervical spine was done and was negative for signs of stroke or cervical spine injury. Magnetic resonance imaging of the spine was negative for spinal cord ischemia. Meanwhile, the patient’s companion shared that a similar episode had occurred one year earlier, when the patient had undergone a computed tomography scan with contrast of the abdomen. The patient had developed bilateral upper and lower extremity weakness after the procedure. She had been taken to the emergency room where neuroimaging was done and was reported as being normal. The patient had been discharged home and the neurological deficit had resolved after a few hours.

Due to the persisting neurological deficit, the patient was admitted to our hospital. The neurologist’s recommendations were that since this was a recurring event and since imaging was negative for pathology, a psychiatric evaluation was to be considered. A psychiatric consultation was requested. During discussions with the psychiatrist, the patient described significant guilt and anxiety in the last few years related to her challenges with immigration and dependency on her extended family and reported feeling very depressed over the last year. The psychiatric evaluation concluded that the patient had baseline anxiety and was suffering from emotional distress and that FNSD cannot be ruled out. The patient was prescribed selective serotonin reuptake inhibitors for depression. When it was shared with the patient that her condition was likely due to a condition called FNSD and that it was not attributed to any neuropathology or neurological disease, she constantly enquired if it was related to the anesthesia.

During her week-long stay at our hospital, minimal improvement in motor function with little movement in the fingers and legs, was noted. She continued to require support for activities of daily living. After one week, she was transferred to a rehabilitation facility where she gradually began to regain function of her upper and lower extremities. She was discharged from the rehabilitation facility three weeks later at which time she was only able to ambulate with a mechanical walker device.

## Discussion

The first description of conversion disorder originated from the ancient Greeks and Egyptians, who observed the condition in women and attributed the symptoms to the uterus [4]. The Greek physician Hippocrates referred to it as hysteria which was derived from the Greek word hysteron meaning uterus. The term conversion disorder originated from Sigmund Freud, who hypothesized that physiologic symptoms affecting motor or sensory functions not explained by organic diseases reflect unconscious conflicts [4]. The term conversion disorder was retained in the 5th edition of the Diagnostic and Statistical Manual of Mental Disorders released in 2013 but was given the subtitle FNSD [3]. The diagnostic criteria were revised in the 5th edition; they covered the same range of symptoms but removed the requirements for a psychological stressor to be present and for feigning to be disproved. A new text revision of the 5th edition of the Diagnostic Manual of Statistical and Mental Disorders that was released in 2022 gave preference to the term FNSD with conversion disorder in parenthesis [3]. A broader range of emotional, cognitive, physical, and social factors are now being described as contributing to FNSD [2]. Malfunction of unconscious predictive systems important to normal movement, sensation and cognition, and neuropsychological abnormalities in cognitive functions such as attention, and sense of control are now being described in relation to FNSD [2].

FNSD is prevalent in up to 0.5% of the general population, with a female to male ratio of 2:1 to 10:1 [4,5,7-9]. Its prevalence in the perioperative setting is unknown [5,9]. Risk factors include 10-35 years of age, low socioeconomic status, personal or family history of a mental health condition, history of a neurological disease that causes similar symptoms, and history of a prior episode suggestive of FNSD [4,10]. The disorder is often associated with family stress, grief, and adjustment difficulties [7]. Increased psychiatric comorbidities such as generalized anxiety disorder, simple phobia, obsessive-compulsive disorder, and major depression are identified in these patients [7]. Patients often report additional associated symptoms, including chronic pain, fatigue, and gut and respiratory symptoms [2].

The etiology is complex with their typically being a precipitating event such as trauma or surgery, against a background of predisposing factors [1]. Preceding psychosocial stressors such as financial problems, relationship difficulties including domestic violence and divorce, and traumatic experiences have been described. The most common functional neurological disorder symptom is loss of motor or sensory function in one or more limbs [1]. Presentation of FNSD occurs largely with the onset and termination of anesthesia and arises most frequently with general anesthesia. Both pediatric and adult populations can be involved [1]. Most cases involve head and neck surgeries, either ear, nose, throat, dental, or ophthalmological cases [6].

The occurrence of FNSD in the postoperative period is increasingly being attributed to the effects of anesthesia with the hypothesis that it arises from the abreactive or dissociative effects of anesthetic agents [1,5]. Additionally, relevant factors may include the pain of the procedure itself, the fear it may induce, and the intense vulnerability of the anesthetized state [1]. Functional imaging findings have suggested a model of aberrant neural circuitry from the prefrontal cortex or cingulate gyrus that may serve as a nexus for inhibitory motor or sensory pathways [4,5]. Thus, certain patients may have a higher susceptibility to FNSD.

In FNSD, the patient’s distress and inability to control their physical symptoms are very real [4]. However, patients may show the lack of concern that should accompany severe physical symptoms [4]. It is essential to carefully eliminate any possible anatomic, physiologic, or pathologic explanations for the neurological deficit and to differentiate FNSD from other psychiatric disorders such as factitious disorders and malingering in which patients feign their symptoms (Table 1) [8-10].

**Table 1:** Differential diagnoses for functional neurological symptom disorder

Organic conditions	Non-organic psychiatric conditions
Residual neuromuscular blockade	Factitious Disorder
Drug intoxication	Malingering
Cerebrovascular Accident	Somatic symptom disorder (somatization disorder)
Intracranial infections	Illness Anxiety disorder
Brain Tumors	Hypochondriasis
Spinal Cord Injury	
Myasthenia Gravis	
Guillain-Barre Syndrome	
Parkinson’s Disease	
Epilepsy	
Multiple Sclerosis	
Polymyositis	
Metabolic Disturbances	
Myopathies (e.g., hypokalemic periodic paralysis)	

Bedside tests may help rule out organic pathology (Table 2) [4,6,7,11].

**Table 2:** Bedside tests that suggest a psychogenic etiology for paralysis of extremities

Bedside tests
Hand-Raise drop test - A positive test occurs when patients avoid hitting themselves when their arm is raised by a provider and then released.
Spinal Injuries Centre Test - Ask the patient to actively flex the knee and then passively flex the knee. The test is positive if the patient cannot actively flex the knee but maintains the passively imposed flexed position. The sensitivity of this test can be improved by talking to distract the patient.
Hoover’s sign - This test is based on the principle of synergistic contraction. If downward pressure is felt from the contralateral heel while raising the non-paralyzed extremity, the weakness is likely psychiatric in nature. Involuntary extension of the pseudo-paralyzed leg also occurs while flexing the non-paralyzed leg.
Abductor Sign - The patient is asked to abduct each leg and this movement is opposed by the hands of the examiner placed on the lateral surface of the patient’s legs. When the paretic leg is abducted, the sound leg stays fixed in organic paresis but moves in the hyper-adducting position in non-organic paresis.
Abductor finger sign - When keeping the fingers abducted in the sound hand against resistance for 2 minutes, involuntary synkinetic finger abduction movements are seen in the paretic hand in functional weakness.
Collapsing or giveaway weakness - The patient’s arm or leg initially provides resistance against an examiner’s touch but then suddenly gives way and does not provide any further resistance.
Tripod sign - Inability to move the upper limbs when in supine or seated position. When the patient is moved to the edge of the bed and placed with arms extended and placed to the sides and behind the torso as in a tripod, there is muscle contraction of bilateral upper extremities and abdominal muscles.
Midline Splitting - Exact splitting of sensation in the midline is said to be a functional sign. Because cutaneous branches of the intercostal nerves overlap from the contralateral side, organic sensory loss should be 1 or 2 cm from the midline.

Unfortunately, a multitude of studies are done, before FNSD is diagnosed. Misdiagnosis is common and may account for one-third of FNSD diagnoses [8].

The patient repeatedly asked her healthcare team the question, “Was this because of the anesthesia.” A discussion of unexpected outcomes with the patient and family is often necessary but the concern for litigation may prove to be a barrier. Discussions should be multidisciplinary, with the involvement of surgery, neurology, psychiatry, and physical therapy specialties. They should provide acknowledgement and assurance that physical symptoms are very real and that the symptoms are likely to improve (Table 3) [4].

**Table 3:** Counseling strategies for patients with functional neurological symptom disorder

Counseling Strategies
Do not inform the patient of the diagnosis on the first postoperative encounter.
Avoid giving patients the impression that there is nothing wrong with them.
Reassurance to patients that symptoms are potentially reversible as there is no structural damage.
Provide examples of conditions that can be stress-related (e.g., elevated blood pressure) when the diagnosis is discussed.
Emphasize that understanding and accepting diagnosis will allow engagement in rehabilitation leading to improvement of the current condition

It is best to avoid discussing that symptoms are purely psychological because it may actually be detrimental and worsen the symptoms [4].

New-onset neurological deficit in the postoperative period is distressing not only for the patient, but also for the medical staff involved in the patient’s care. Members of the healthcare team may suffer from apprehension, depression, and fear of litigation. A second victim phenomenon occurs when healthcare providers experience emotional or physical distress as a result of traumatic clinical events [12]. Support programs may help second victims navigate the post-event experience and offload associated emotional labor.

The first step in the management of FNSD is to provide patients with a name for their likely or suspected diagnosis [13]. With FNSD being a disabling condition at the intersection of neurology and psychiatry, advances are being made in finding evidence-based treatments [14]. The rate of associated depression has been reported as 20-40% with the rate of reported anxiety being about 40% [13]. Treatment involves psychotherapy, elucidating stressors, and improving the patient’s ability to cope with the aforementioned stressors. In addition to a psychological model, ongoing research has introduced a neurobiological perspective. Dysfunction of brain networks including attention and perception has been described which would influence management [13]. Most cases are self-limiting and improve over time. Symptoms recur in 20-25% of patients within one year [10]. Physical therapy is essential in patients with prolonged symptoms to prevent sequelae such as muscle weakness [4]. Pharmacologic treatment is directed at co-existing psychological disorders [4,9]. At present few well-validated outcome measures exist to assess treatments, with more research being needed [15]. Outcome measures that are currently recommended include the clinical global measurement-improvement scale for core symptoms, Hamilton rating scales for anxiety and depression, short-form health survey-36 for quality of life, work and social adjustment scale for general functioning, and determining the extent of health care contacts to assess health care resource use [15]. Development of preoperative tools that screen for known risk factors, associated psychological co-morbidities, and psychosocial stressors, may help identify patients at risk.

## Conclusions

FNSD is a disabling condition. An anesthesia model has been suggested, with the helplessness associated with the anesthetized state being associated with evoking previous traumatic experiences. The possible dissociative effects of anesthetic agents are also being implicated. The occurrence of FNSD in the postoperative period is associated with clinically significant impairments in the patient’s functional, social, and occupational life. Patients may endure prolonged hospitalization, with increased utilization of hospital resources. Preoperative psychological assessment may help identify patients at risk for FNSD. If postoperative neurological deficit occurs, FNSD should be on the list of differential diagnoses, despite the depth of sedation or type of anesthetic utilized. Detailed neurological, metabolic, and psychiatric assessments should be done with FNSD being a diagnosis of exclusion. Management should comprise honest disclosure, reassurance of recovery, and reinforcement of alternative coping strategies. More research is needed to quantify outcome measures. Counseling should be provided and follow-up with a psychiatrist or psychologist should be scheduled. The development of screening tools may help identify patients at risk.
